# A Case of Rupioid Syphilis Masquerading as Aggressive Cutaneous Lymphoma

**DOI:** 10.4084/MJHID.2015.026

**Published:** 2015-04-20

**Authors:** Jonathan Braue, Thomas Hagele, Abraham Tareq Yacoub, Suganya Mannivanan, Lubomir Sokol, Frank Glass, John N. Greene

**Affiliations:** 1 University of South Florida Morsani College of Medicine, 12901 Bruce B. Down Blvd, Tampa, Florida 33612-4742; 2 University of South Florida Morsani College of Medicine, Department of Dermatology and Cutaneous Surgery, 12901 Bruce B. Down Blvd, Tampa, Florida 33612-4742; 3 Moffitt Cancer Center, 12902 Magnolia Drive, Tampa, Florida 33612-9497; 4 University of South Florida, Morsani College of Medicine, Division of Infectious Disease and International Medicine, 1 Tampa General Circle, G323, Tampa, Florida 33612-9497; 5 Moffitt Cancer Center, Department of Cutaneous Oncology, 12902 Magnolia Drive, Tampa, Florida 33612-9497; 6 Moffitt Cancer Center, University of South Florida College of Medicine, 12902 Magnolia Drive, Tampa, Florida 33612-9497

## Abstract

Secondary syphilis has been known since the late 19th century as the great imitator; however, some experts now regard cutaneous lymphoma as the great imitator of skin disease. Either disease, at times an equally fastidious diagnosis, has reported to mimic each other even. It is thus vital to consider these possibilities when presented with a patient demonstrating peculiar skin lesions. No other manifestation of secondary syphilis may pose such quandary as a rare case of rupioid syphilis impersonating cutaneous lymphoma. We present such a case, of a 36-year-old HIV positive male, misdiagnosed with aggressive cutaneous lymphoma, actually exhibiting rupioid syphilis thought secondary to immune reconstitution inflammatory syndrome (IRIS).

## Introduction

In 1859, the French dermatologist Pierre Bazin first used the term malignant to describe a case of secondary syphilis, and five years later, in a thesis, his student Dubue defined malignant syphilis.^1^ Furthermore, in 1896, at the Third International Congress of Dermatology in London, the Danish dermatologist Haslund and German dermatologist Neisser, independently classified malignant syphilis as a rare and ulcerating form of secondary syphilis and not an early form of tertiary syphilis as was previously contemplated.^1^–^3^ Malignant syphilis, also referred in the literature as syphilis maligna praecox, lues maligna, or rupioid syphilis, is defined to present with pleomorphic multiple round to oval papules, papulopustules, or nodules with ulceration, without central clearing, and additionally exhibiting a lamellate brown to black rupioid crust.^4^,^5^ The term rupioid stems from the rupia, or “oyster-like” appearance of these lesions, and, therefore, is the author’s preferred title of this condition. Oddly, diagnosis by skin biopsy in affected patients proves difficult. In general, there is an extreme paucity of spirochetes in the skin lesions and special staining and dark field microscopy may not lead to a histologic diagnosis.^5^ To guide the clinical diagnosis, Neisser postulated five characteristics of rupioid syphilis (Table 1).^1^ Moreover, in 1969, Fisher et al proposed four additional criteria in order to identify this rare variant (Table 2).^5^ This particular expression of secondary syphilis was quite rare prior to the advent of HIV, with an estimated frequency of 0.12–0.36 % and only about two dozen cases published in the English literature prior to 1994.^2^ Since the beginning of the HIV epidemic, the incidence of rupioid syphilis has been steadily rising, making it a disease of vital recognition for any patient with suspicious cutaneous lesions.^2^

Only cutaneous lymphoma, with its myriad presentations both clinically and histologically, could be such a comparably challenging diagnosis as that of secondary syphilis. In 1975, Abell et al, gathered histologic data on skin biopsies from 57 patients with serologically proven secondary syphilis.^6^ The most common clinical diagnosis previous to histologic examination from these biopsies, other than syphilis, was said to be in order of frequency, pityriasis lichenoides, psoriasis, eczema, insect bites, sarcoidosis, leishmaniasis, and lymphoma.^6^ Moreover, many reports of syphilis mimicking mycosis fungoides (MF) have been documented. In 1980, Levin et al described a patient with compelling evidence for the diagnosis of MF, only to later be revealed as a strange case of secondary syphilis.^7^ Since Levin’s report, there have been other documented cases of secondary syphilis mimicking MF.^8^–^10^ Our patient highlights similar points, and, in addition, offers an etiology to the cause of rupioid syphilis in HIV underlining the importance of a detailed medical history and adequate laboratory tests in an HIV infected patient.

## Case Report

In late July, a 36-year-old African American man with a history of HIV and remote history of properly treated syphilis, presented to our institution for a second opinion regarding his recent diagnosis of peripheral T-cell lymphoma, not otherwise specified (PTCL-NOS). The patient’s history revealed he had not been on anti-retroviral therapy (ART) for many years due to adverse effects. During the evaluation by his outside practitioner, he was told that his viral load was “very high”, but uncertain in the number, though his CD4 count was 325 cells/ml. He was thus started on a combination of elvitegravir, cobicistat, tenofovir, and emtricitabine. Two weeks after restarting ART, he reported fevers, chills, night sweats and several erupting skin lesions on his jaw, scalp, nose, legs, and arms. Follow-up CD4 count revealed a moderate increase up to 450 cells/ml.

In June, during his initial work-up at an outside facility, a skin biopsy was done on a right scalp lesion and revealed an atypical lymphohistiocytic infiltrate in the subcutaneous tissue, consisting mainly of CD3+, CD5+, CD7+, and CD8+ cells. Histiocytes were positive for CD68 marker, and the T-cell receptor (TCR) gene was clonally rearranged. Successively, a computed tomography (CT) scan of his neck, chest, and abdomen demonstrated several prominent lymph nodes in the neck, axillary, and inguinal areas, ranging from 14 mm up to 19 mm. A biopsy of the terminal ileum and of a rectal mass, which revealed on the former CT scan, were performed. The pathology of the terminal ileum showed an atypical submucosal lymphoid infiltrate consisting of 50% CD8+ and 50% CD4+ cells. The biopsy of the rectal mass showed atypical lymphoid cells in a necrotic background. The bone marrow biopsy was performed and showed normocellular marrow (40–60% cellularity) with regular trilinear hematopoiesis. No abnormal lymphoid population was detected using flow cytometry, and cytogenetics was normal 46, XY. Further inguinal and peripheral lymph node biopsies failed to show evidence of lymphoma. The differential at this time consisted of PTCL NOS versus CD8+ aggressive epidermotropic cytotoxic T-cell lymphoma. The patient underwent port placement and was to begin chemotherapy. It was at this time that he presented to us.

**Figures 1 and 2:**
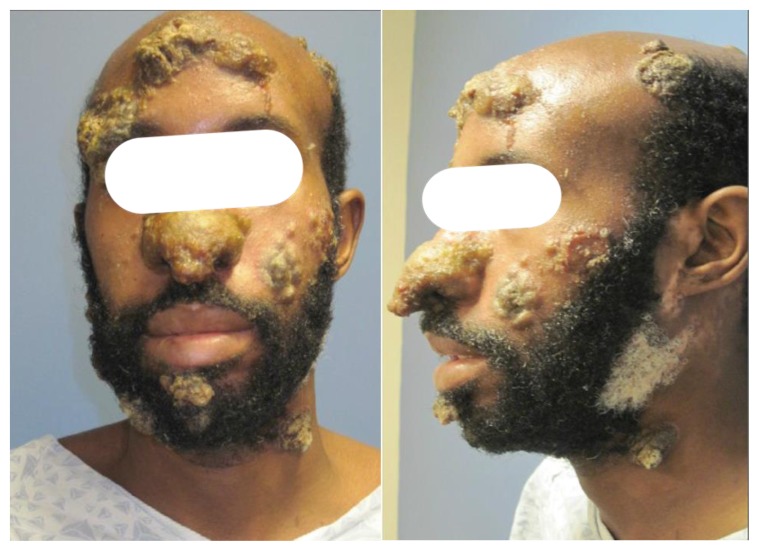
There are several large one to two inch, discrete, yellowish-brown, necrotic crusted malodorous verucous plaques and tumors.

**Figure 3:**
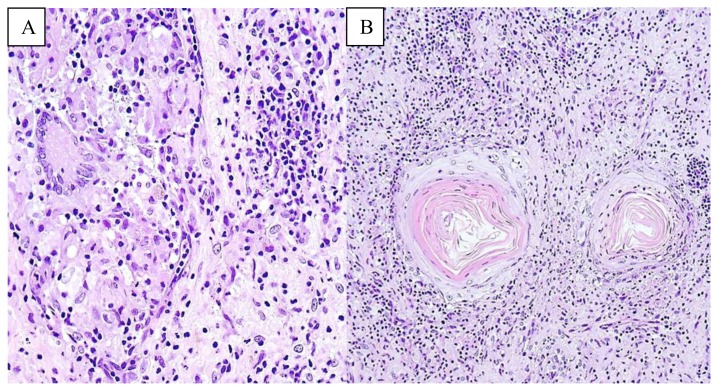
Skin biopsy from anterior scalp lesion. H+E Stain. **A:** Prominent dermal infiltrate of epithelioid histiocytes. Poorly formed granulomas with Giant cells are present. **B:** A dermal perifollicular lymphohistiocytic infiltrate is present. The lymphocytes are predominantly small with mildly irregular nuclear contours.

On initial presentation, the patient exhibited on his nose, forehead, scalp, cheek, neck, and bilateral upper and lower extremities, several large one to two inch, discrete, yellowish-brown, necrotic crusted malodorous verrucous plaques and tumors (Figures 1 and 2). He reported that the large plaque on his nose was occluding his nares and draining a foul-smelling brownish fluid, making it difficult for him to breath. At this time, he was not experiencing fever, chills, or night sweats, but did continue to endorse some unintentional weight loss. The lesion located on his right arm was quite painful, making it difficult to sleep, but there were no intraoral lesions present. At this time, further skin biopsies were performed. The pathology (Figures 3A and B) showed an atypical lymphohistiocytic infiltrate that appeared to be reactive without clonal etiology. There were areas of necrosis and no discrete granulomas. The epidermis showed spongiosis, keratinocyte necrosis; acute inflammatory infiltrate, and pseudo-epitheliomatous hyperplasia with flow cytometry of the skin specimen showed predominantly small mature lymphocytes and histiocytes with no clonal findings and no evidence of T-cell lymphoma. Acid-fast bacilli (AFB), cytomegalovirus, herpes simplex and varicella zoster viruses, and Warthin-Starry staining were negative. AFB, fungal, and bacterial cultures were also negative. Throughout this work-up, the patient reported that his lesions were only getting worse, and his nasal breathing problems were also increasing.

After effectively ruling out cutanous lymphoma, secondary syphilis of the rupioid type became top of our differential. The fluorescent treponemal antibody (FTA-ABS) was positive, and the rapid plasma reagin titer was very highly reactive at 1:1,024. Cerebrospinal fluid (CSF) studies were negative. In conjunction with the time course and increase in his CD4 count after starting ART, it was thought that his rupioid syphilis was derived from an immune reconstitution inflammatory syndrome (IRIS). He was consequently commenced on intravenous penicillin G (IV) 24 million units daily but after about 24 hours from initiation, he developed fever and chills. It was unclear if this was due to an allergic reaction or secondary to a mild Jarisch-Herxheimer reaction, which is expected during treatment for rupioid syphilis. However, the dose was decreased and within days, the lesions began resolving and the patients breathing improved. He was treated in this manner for two weeks and then discharged with an additional three weekly injections of intramuscular benzathine penicillin 2.4 million units. The crusted lesions flaked off within a week of treatment. Within several weeks of treatment, all of the cutaneous lesions were resolved, but the patient was subsequently lost to follow-up without reevaluation of his intra-abdominal lesions.

## Discussion

Since the beginning of the 20th century, when Neisser described 31 patients with rupioid syphilis and devised clinical criteria to aid in the diagnosis of this ailment, until the early 1900’s only a handful of cases were reported.^2^,^4^ During this period, rupioid syphilis classically afflicted those who were malnourished, alcoholic, or injection users.^5^,^8^ However, since the emergence of the HIV-era, the incidence of syphilis, in general, has been increasing, and with it, the incidence of rupioid syphilis.^4^,^5^,^8^ This particular manifestation of the ancient Treponema remains rare and thus difficult to recognize and diagnose, especially when disguised so persuasively as another disorder like cutaneous lymphoma.

There have been many reports of unusual presentations of secondary syphilis mimicking cutaneous lymphoma, both clinical and histologically.^6^–^10^ To our knowledge, there is only a single case that reports a patient with rupioid syphilis masquerading as mycosis fungoides (MF).^8^ However, our case proves unique in several key points. First, differing from all of the previous reports, it is not an indolent form of cutaneous lymphoma like MF that our patient was thought to posses, but rather a very aggressive form in need of systemic chemotherapy.

Secondly, and somewhat curiously, the initial TCR gene rearrangement on our patient did indicate a clonal T-cell proliferation unlike what was reported in previous cases. Finally, we propose an etiology to our patient’s eruption of rupioid syphilis, in that it could have been secondary to an immune reconstitution inflammatory syndrome (IRIS).

Prior to the 1970’s it was not uncommon for secondary syphilis to be clinically confused with MF.^6^,^11^ In 1975, Abell et al described several different histologic appearances in their biopsies, and proposed that the presence of a heavy upper dermal infiltrate with invasion of mononuclear cells into the epidermis would simulate MF.^6^ They then argued however, that the usual presence of plasma cells and lack of large hyperchromatic mononuclear cells with crenate nuclei would speak against a diagnosis of MF and support secondary syphilis. Yet, there was a single case where a small population of large hyperchromatic mononuclear cells was present amongst a lymphohistiocytic infiltrate, thus at first glance being very easily misconstrued as cutaneous lymphoma.^6^ Moreover, several other cases since Abell’s have made equally compelling histological arguments for a diagnosis of MF.^7^–^10^ However, when reported, the T-cell receptor (TCR) gamma/beta gene rearrangement assay was negative, and in some of the cases the Warthin-Starry stain demonstrated spirochetes.^8^,^9^ In our patient, MF was not a consideration based on the rapidity of the eruption and more aggressive pathology seen from the skin biopsies. For this reason, it is vital to consider alternative diagnosis and repeat of key laboratory work-up prior to initiating highly toxic chemotherapy. Why then did out patient’s skin express such an aggressive pathology, and why was the initial peripheral flow cytometry indicative of a clonal T-cell expansion? Perhaps it was truly related to a rebound hunger from his immune system following initiation of ART and thus an underlying IRIS.

IRIS is a paradoxical immunological phenomenon, which occurs in patients whom are recovering from an immunocompromised state.^12^ In the HIV affected individual, this can occur in up to 25% within a few months of being placed on ART, with additional risk incurred when placed on ART late in the disease course.^12^–^15^ Attempts have been made to tabulate clinical criteria to diagnose IRIS, as there are no currently accepted laboratory data that can render a diagnosis. In 2004, French et al decreed a set of criteria for this purpose.^13^ Our patient met the minimum criteria for a diagnosis of IRIS based on French et al’s publication, on the basis that he did respond to antiretroviral therapy, he did have a very atypical presentation of his infection, his CD4+ count raised, and the increase in the immune response was directed at his syphilis. Moreover, the atypical lymphocytic infiltrate seen in his skin, a diagnostic criterion, could certainly be the reason for the aggressive cutaneous lymphoma mimicry. Additionally, French et al and Martin-Blondel et al discuss risk factors for development of IRIS, and although our patient’s CD4 count was not critically low prior to the induction of ART, many of the other risk factors were met, such as the high pre-ART viral load, strong response to ART based on increase in CD4 counts, ART-naïve patient, black ethnicity, and his subclinical infection.^13^,^16^

Furthermore, most recently the consensus criteria for the diagnosis of IRIS were put forth by the International Network for the Study of HIV-associated IRIS (Table 3).^12^ Our patient met many of these criteria, and although he did not have follow-up HIV RNA titers, there was a clear response to treatment as his CD4 count increased by nearly 175 cells/ml. It is thus very likely that our patient did suffer from IRIS after initiating ART, which could have led to the initial positive peripheral flow cytometry and the peculiar pathology demonstrated from his multiple biopsies.

## Conclusion

Because of the similar appearance of rupioid syphilis and cutaneous lymphoma, this case validates the notion of truly combining the clinical and pathologic information to distinguish the two entities. Our patient could have significantly worsened if he had been placed on chemotherapy rather than properly treated with antibiotics. Additionally, through a diagnosis of IRIS, we suggest a plausible relationship between our patient’s rare presentation of rupioid syphilis and the imitation that took place.

## Figures and Tables

**Table 1:** Five characteristics of rupioid syphilis postulated by Neisser

**1**	The disease has a relatively short incubation period
**2**	Constitutional symptoms are pronounced
**3**	The skin and frequently the mucous membranes of the mouth and nose present multiple irregularly distributed lesions consisting of large pustules, ulcers, and rupioid ecthymatous lesions
**4**	The patient may have characteristics of the milder forms of the disease such as mucous membrane buccal patches, etc.
**5**	The skin lesions are pleomorphic and demonstrate papulopustules, beginning ulcerations, deep ulcerations, ulcers covered with crusts, and healing lesions

**Table 2:** Four additional criteria proposed by Fisher in order to identify this rare variant.

**1**	Compatible gross and microscopic morphology
**2**	A high titer serologic test for syphilis
**3**	Jarisch-Herxheimer reaction
**4**	Dramatic response to antibiotic therapy

**Table 3:** Modified from the International Network for the Study of HIV-Associated IRIS.

Consensus Criteria of Immune Reconstitution Inflammatory Syndrome: Case Definition	
**1**	Response to antiretroviral therapy by Receiving HIV antiretroviral therapy and Virologic response with > 1log 10 copies/mL decrease in HIV RNA (if available)
**2**	Clinical deterioration of an infectious or inflammatory condition temporally related to antiretroviral therapy initiation
**3**	Symptoms can not be explained by expected clinical course of a previously recognized and successfully treated infection. medication side effect or toxicity treatment failure, and complete non-adherence
